# The Activity of *Cotinus coggygria* Scop. Leaves Extract on *Staphylococcus aureus* Strains in Planktonic and Biofilm Growth Forms

**DOI:** 10.3390/molecules21010050

**Published:** 2015-12-30

**Authors:** Katarína Rendeková, Silvia Fialová, Lucia Jánošová, Pavel Mučaji, Lívia Slobodníková

**Affiliations:** 1 Department of Pharmacognosy and Botany, Faculty of Pharmacy, Comenius University in Bratislava, Odbojárov 10, 832 32 Bratislava, Slovakia; rendekova18@uniba.sk (K.R.); mucaji@fpharm.uniba.sk (P.M.); 2 Department of Pharmaceutical Analysis and Nuclear Pharmacy, Toxicological and Antidoping Center, Faculty of Pharmacy, Comenius University in Bratislava, Odbojárov 10, 832 32 Bratislava, Slovakia; veizerova@fpharm.uniba.sk; 3 Institute of Microbiology of the Medical Faculty and the University Hospital in Bratislava, Comenius University in Bratislava, Sasinkova 4, 811 08 Bratislava, Slovakia; livia.slobodnikova@fmed.uniba.sk

**Keywords:** *Cotinus coggygria*, leaves, fingerprint, antibacterial activity, anti-biofilm activity, *Staphylococcus aureus*

## Abstract

The purpose of this study was to detect the effectiveness of *Cotinus coggygria* Scop. leaves methanol extract against planktonic and biofilm growth forms of *Staphylococcus aureus*. The antimicrobial activity was determined by the broth microdilution test. Minimal inhibitory concentrations and minimal bactericidal concentrations were detected against two collection and ten clinical *S. aureus* strains. Anti-biofilm activity of the tested extract was detected using 24 h bacterial biofilm on the surface of microtiter plate wells. The biofilm inhibitory activity was evaluated visually after 24 h interaction of extract with biofilm, and the eradicating activity by a regrowth method. The tested extract showed bactericidal activity against all *S. aureus* strains (methicillin susceptible or methicillin resistant) in concentrations ranging from 0.313 to 0.625 mg·mL^−1^. Biofilm inhibitory concentrations were 10-times higher and biofilm eradicating concentrations 100-times higher (8 and 32 mg·mL^−1^, respectively). The phytochemical analysis of *C. coggygria* leaves 60% methanol extract performed by LC-DAD-MS/MS revealed quercetin rhamnoside, methyl gallate, and methyl trigallate as main constituents. Results of our study indicate that *C. coggygria*, rich in tannins and flavonoids, seems to be a prospective topical antibacterial agent with anti-biofilm activity.

## 1. Introduction

Growing antimicrobial resistance is one of the most discussed problems in recent times. An enormous effort in today’s medicine is focused towards the concept of slowing or even interrupting the process of antimicrobial resistance development and its overall spread. The worldwide spread of resistance and ineffectiveness of antimicrobial therapy caused by multidrug-resistant pathogenic microorganisms present a serious threat to human population [1,2]. Among them, *Staphylococcus aureus* plays an important role. This bacterium can colonize the skin and mucosal surfaces of animals and humans and belongs to the most frequent and important human pathogens, threatening particularly patients with broken primary protective barriers (skin and mucosa), immuno-compromised people, and people with indwelling or implanted medical devices. This bacterial species is one of the most frequent causative agents of skin and soft tissue infections, and one of the major agents of nosocomial infections. Strains resistant to methicillin (MRSA), and to the other antimicrobial drugs are especially dangerous [3].

Mainstream medicine is increasingly receptive to the use of antimicrobials and the other drugs derived from plants [2]. Plants produce secondary metabolites with various chemical compositions and different biological effects, including antimicrobial activity against the pathogenic microorganisms. *Cotinus coggygria* Scop., also known as a “Smoke tree“, fustic or sumac is an ornamental plant from the family Anacardiaceae. Geographic distribution of this plant starts in Southern Europe and continues through the Caucasus to the central China and the Himalayas. In the traditional medicine of various countries, *C. coggygria* has been used for its anti-inflammatory, antimicrobial and wound-healing properties. This plant is mainly employed to treat injuries of the skin and mucosal tissues (buccal, gastric, intestinal); other uses include hepatobiliary disorders, hepatitis, and fever reduction. These indications are supported by the content of tannins, essential oils and various flavonoids. Polyphenols from *C. coggygria* are represented by gallic acid and its derivatives such as methyl gallate, pentagalloyl glucose with antioxidative effects as well as by catechin, procyanidins and flavonoids [4,5,6,7]. Nowadays, interest in the antibacterial activity of this medicinal plant has been renewed [8]. The *in vitro* antimicrobial activity of stems, shoots or leaves of *C. coggygria* was examined on different bacterial species, among them on *Staphylococcus aureus*, *Bacillus subtilis*, *Klebsiella pneumoniae*, *Escherichia coli*, *Micrococcus lysodeicticus*, *Pseudomonas aeruginosa*, *Enterococcus faecalis* [9,10,11]. However, there is little evidence of the bactericidal activity of these plant extracts, and lack of information about effect of *C. coggygria* leaves extracts on clinical *S. aureus* strains, including MRSA, and about their antibiofilm activity.

The aim of this study was to examine the *in vitro* activity of a 60% methanol extract of *C. coggygria* leaves on collection and clinical *S. aureus* strains. In this work, the effect of the analyzed extract on biofilm form of *S. aureus* was detected as well. Phenolic fingerprint using LC-DAD-MS/MS method was performed for better understanding which compounds present in the tested extract that could be involved in the antimicrobial activity.

## 2. Results and Discussion

### 2.1. Antimicrobial Activity

The activity of the 60% methanol extract of *C. coggygria* leaves was tested against two collection *S. aureus* strains (CCM 4750 corresponding to ATCC 4330, and CCM 4223 corresponding to ATCC 29213) and ten clinical isolates originating from skin lesions swabs. Three clinical *S. aureus* strains were resistant to oxacillin/methicillin, four to erythromycin, three to clindamycin, three to gentamicin, four to ciprofloxacin, and one to tetracycline and chloramphenicol. Four strains were resistant to three or more tested antibiotics. The methanol extract of *C. coggygria* leaves had antibacterial activity against all *S. aureus* strains, without respect to the patterns of their antimicrobial resistance, including all MRSA strains. The minimal inhibitory concentrations (MIC) ranged from 0.156 to 0.313 mg·mL^−1^. The extract killed all bacterial strains in concentrations from 0.313 to 0.625 mg·mL^−1^ (MBC; see Table 1).

In previous studies on *C. coggygria* the antibacterial activity was tested using extracts prepared by various methods (*i.e.*, with various qualitative and quantitative content of active compounds), and various experimental procedures [8]. In our study, 60% methanol extract of *C. coggygria* leaves was used to determine the minimal inhibitory and minimal bactericidal concentrations on the tested *S. aureus* strains by a standardized broth microdilution method. The main compounds of the extract were quercetin rhamnoside, methyl gallate, and methyl trigallate. Quercetin is considered to be a strong antioxidant agent. Furthermore, Hirai *et al.* revealed the unique antibacterial properties of quercetin against *S. aureus*, including MRSA [12]. Antibacterial activity of methyl gallate is also well documented [13]. Our results of bacteriostatic activity are comparable with those obtained by Matić *et al.*, who tested a *C. coggygria* stem methanol extract against *S. aureus*, *Bacillus subtilis*, *Klebsiella pneumoniae*, *Escherichia coli*, *Micrococcus lysodeicticus* and *Candida albicans*, using the broth macrodilution test (the obtained MIC for the *S. aureus* strain was equal to 250 μg·mL^−1^) [9]. Similar results were obtained by Marćetić *et al.* [11] against staphylococci with chloroform and water fractions of *C. coggygria* young shoots extract tested by the broth microdilution method (MIC 100 μg·mL^−1^), and even better results with acetone and ethyl acetate fractions (MIC equal to 25 μg·mL^−1^). The bactericidal concentrations of our extract against the tested *S. aureus* strains were similar or one- to two-dilutions higher than the MICs; we did not find any data about the MBCs obtained by other research groups. Our results indicate that in addition to the inhibitory effect, the *C. coggygria* leaves methanol extract eradicated bacteria in the tested samples at reasonable concentrations.

**Table 1:** Antimicrobial activity of *C. coggygria* leaves 60% methanol extract on *Staphylococcus aureus* strains.

Strain	Origin	Resistance to	MIC (mg·mL^−1^)	MBC (mg·mL^−1^)
1	Atopic dermatitis	GEN, CIP	0.313	0.313
2	Atopic dermatitis	ERY, CLI, GEN	0.313	0.625
3	Atopic dermatitis		0.313	0.625
4	Atopic dermatitis	OXA, ERY, CLI, CIP	0.313	0.313
5	Atopic dermatitis	ERY, CLI, GEN	0.156	0.625
6	Impetigo		0.313	0.313
7	Impetigo		0.313	0.313
8	Impetigo	OXA, CIP	0.313	0.313
9	Impetigo	OXA, CIP, TET, CMP	0.313	0.313
10	Impetigo	ERY	0.156	0.313
11	CCM 4750 (MRSA)	n.a.	0.156	0.313
12	CCM 4223 (MSSA)	n.a.	0.156	0.313

MRSA—methicillin resistant *S. aureus*; MSSA—methicillin susceptible *S. aureus*; MIC—minimal inhibitory concentration; MBC—minimal bactericidal concentration; CCM—Czech Collection of Microorganisms; OXA—oxacillin (methicillin); ERY—erythromycin; CLI—clindamycin; GEN—gentamicin; CIP—ciprofloxacin; TET—tetracyclin; CMP—chloramphenicol; n.a.—not applicable.

### 2.2. Antibiofilm Activity

The biofilm inhibitory and biofilm eradicating activity of *C. coggygria* leaves methanol extract was evaluated on 24 h *S. aureus* biofilm, produced on the surface of polystyrene microtiter plate wells. Biofilm forming activity was detected in microtiter plate wells [14]. Intensity of biofilm production was detected spectrophotometrically at 570 nm after staining with crystal violet. All strains were good biofilm producers—they built biofilms corresponding to absorbance values from 0.397 to 2.255. Two *S. aureus* strains (No. 3 and 11; see Table 1) were selected for our anti-biofilm activity study. The selection criterion was the massive biofilm formation activity of the clinical strain No. 3 and the resistance to methicillin of the collection strain CCM 4750. The concentration of extract able to suppress multiplication of bacteria in biofilms (the minimal biofilm inhibitory concentration; MBIC), detected after 24 h interaction with biofilm-associated bacteria was in both cases 8 mg·mL^−1^. The concentration which killed bacteria in biofilm (the minimal biofilm eradicating concentration; MBEC) was detected after additional 24 h cultivation of biofilm with extract-free medium. The measured values were in both strains 32 mg·mL^−1^ (Table 2).

**Table 2:** Antibiofilm activity of *C. coggygria* leaves 60% methanol extract on selected *S. aureus* strains.

Strain	Biofilm Producing Activity (A_570_)	MBIC (mg·mL^−1^)	MBEC (mg·mL^−1^)
3	2.255	8	32
11	0.614	8	32

MBIC—minimal biofilm inhibitory concentration; MBEC—minimal biofilm eradicating concentration; A_570_—absorbance of crystal violet-stained 24 h biofilm at 570 nm.

Biofilm production is an important virulence factor of microorganisms connected with chronic infections, such as sinusitis, otitis media, cholecystitis, prostatitis, osteomyelitis, or chronic skin and wound infections, as well as infections associated with foreign bodies (implants and catheters), with *S. aureus* as a frequent agent of these infections [15]. The unfavorable consequence of biofilm formation around bacteria in the infectious focus is their increased resistance to antimicrobial therapy and mechanisms of immunity, as a result of different transcriptome expression in comparison with planktonic bacteria, leading to an adaptive form of bacterial resistance [16]. Despite numerous reports about various biological activities of *C. coggygria* extracts, there is a lack of information about their anti-biofilm activity. In the present study we demonstrated for the first time the suppressive and eradicating activity of *C. coggygria* leaves methanol extract on already established, 24 h *S. aureus* biofilms. The test was performed *in vitro* with biofilms on polystyrene surface, which are widely used for the detection of anti-biofilm activity of various antimicrobial agents [17,18,19]. The biofilm inhibitory concentrations were approximately 10-times higher than the minimal inhibitory concentrations, and the eradicating concentrations were even 100-times higher. This phenomenon is well known from tests with anti-biofilm activity of antibiotics [20,21]. Despite of the relatively high MBIC and MBEC of methanol *C. coggygria* leaves extract tested in our study, it could be a promising local anti-biofilm agent for a complex therapy of chronic skin and wounds infections. However, further studies are needed to evaluate its biocompatibility and harmlessness during therapeutic usage.

### 2.3. Phenolic Fingerprint of the 60% Methanol Extract of C. coggygria Leaves

Phenolic compounds were identified by LC-MS/MS, using authentic standards and database searches by comparing the mass spectra with a maximum allowed mass deviation of 10 ppm. We detected the presence of 16 phenolic compounds, tannins and flavonoids summarized in Table 3 along with their retention times (TR), observed mass in negative ionization mode, MS/MS fragment ions of each secondary metabolite as well as bibliographic references used in the characterization process. The compounds detected in the analyzed extract were characterized by means of MS data, together with the interpretation of the observed MS/MS spectra in comparison with data listed in the available literature. Peak 1, which presented a pseudomolecular [M − H]^−^ ion at *m*/*z* 191.0564 with a product ion at *m/z* 173.0458 was identified as quinic acid. Compound 2 (TR 6.59) with the precursor ion [M − H]^−^ at *m*/*z* 331.0681 and product ion at *m*/*z* 169.0153 [M − H − 162]^−^ has been assigned to galloyl hexose. Peak 3, with [M − H]^−^ at *m*/*z* 169.0147 was identified as gallic acid, compared with an authentic standard. Peak 4 showed [M − H]^−^ at *m*/*z* 325.0577 with a major fragment ion at *m*/*z* 169.0148 indicating a neutral loss of a shikimate moiety [M − H − 156]^−^ revealing the presence of galloylshikimic acid. Peak 5 with [M − H]^−^ at *m*/*z* 315.0313 and product ion at *m*/*z* 153.0198 [M – H − 162]^−^ was identified as protocatechuic acid hexoside. A dominant peak 6 with the precursor [M − H]^−^ ion at *m*/*z* 183.0300 was assigned to methyl gallate. The related peaks 15 and 16 with [M − H]^−^ at *m*/*z* 335.0416 and 469.0533 resp. both showed product ions at *m*/*z* 183 and were identified as methyl digallate and methyl trigallate, respectively. The fragment ions at *m*/*z* 321.0267 and at *m*/*z* 169.0155 point to the loss of a gallic acid moiety from the precursor ion [M − H]^−^ at *m*/*z* 473.0369 which led to the identification of trigallic acid. The precursor [M − H]^−^ ions at *m*/*z* 635.0928, 787.1034 and 939.121 corresponding to a neutral loss of a galloyl moiety [M – H − 152]^−^ and a neutral loss of glucose [M − H − 162]^−^ were identified as tri- , tetra—and pentagalloyl hexoside respectively. In the investigated extract we also detected a presence of flavonoids. They were represented by myricetin and quercetin derivatives; myricetine glucoside with [M − H]^−^ at *m*/*z* 479.0849 and myricetin rhamnoside with [M − H]^−^ at *m*/*z* 463.0903, both with myricetin product ions at *m*/*z* 316.0205. Peak 14 was identified as quercetin glucoside with [M − H]^−^ at *m*/*z* 463.0901 and a typical loss of glucose [M – H − 162]^−^. Peak 17 was assigned to quercetin rhamnoside with [M − H]^−^ at *m*/*z* 447.0956 and was found to be as the most abundant phenolic compound in the investigated extract. All mentioned substances were previously recorded in the literature [22,23], but not all of them in *C. coggygria*, as only gallic acid, methyl gallate, pentagalloyl hexoside, tetragalloyl hexoside and myricetin and quercetin as aglycones were identified in different parts of *C. coggygria* in the past [5,8,24,25].

**Table 3:** Polar phenolic compounds in *C. coggygria* leaves methanol extract, their corresponding retention times (T_R_), molecular ions [M − H] and MS^2^ fragments in LC-MS analysis and quantitative abundance of polar phenolic compounds (μg·mg^−1^).

Peak No.	Compound	T_R_ (min)	[M − H]^−^ (*m*/*z*)	MS^2^ (20 eV) (*m*/*z*)	Mass Concentration (μg·mg^−1^) * ± SD	Reference
1.	Quinic acid	5.019	191.0564	173.0458	2.1 ± 0.01 ^#^	[22]
2.	Galloyl hexose	6.599	331.0681	191.0576, 169.0153	1.9 ± 0.01 ^#^	[22]
3.	Gallic acid	7.521	169.0147	125.0251	9.8 ± 0.01 ^#^	[5,11]
4.	Galloylshikimic acid	8.600	325.0577	169.0148	1.5 ± 0.01 ^#^	[22]
5.	Protocatechuic acid hexoside	12.314	315.0313	153.0198, 109.0304	1.3 ± 0.08 ^#^	[22]
6.	Methyl gallate	21.083	183.0300	140.0106, 124.0171	31.2 ± 0.03 ^#^	[5]
7.	Trigalloyl hexoside	30.510	635.0928	465.0673, 169.0150	<LOQ ^#^	[22]
8.	Tetragalloyl hexoside	31.774	787.1034	635.0890, 617.0881, 393.0474	1.3 ± 0.10 ^#^	[22,25]
9.	Tetragalloyl hexoside	32.300	787.1033	635.0898, 617.0776, 393.0458	<LOQ ^#^	[22,25]
10.	Myricetin glucoside	33.090	479.0849	316.0205, 317.034	2.7 ± 0.09 ^†^	[22]
11.	Trigallic acid	33.538	473.0369	321.0267, 169.0155	<LOQ ^#^	[22]
12.	Pentagalloyl hexoside	35.302	939.121	787.1022, 617.0859, 469.0534, 393.0480	<LOQ ^#^	[5]
13.	Myricetin rhamnoside	36.724	463.0903	316.0206	8.1 ± 0.09 ^†^	[22,23]
14.	Quercetin glucoside	37.435	463.0901	301.0007	2.5 ± 0.29 ^†^	[22]
15.	Methyl digallate	38.304	335.0416	183.0303	<LOQ _#_	[22]
16.	Methyl trigallate	38.910	469.0533	335.0423, 183.0309	29.7 ± 4.39 ^#^	[22]
17.	Quercetin rhamnoside	45.941	447.0956	301.0206	35.3 ± 2.09 ^†^	[22,23]

* Values (μg·mg^−1^ dry extract) are presented as means ± standard deviation (*n* = 3), External standards: gallic acid ^#^ and quercetin ^†^; LOQ—limit of quantification.

### 2.4. The Quantification of Phenolics

From Table 3 it is evident that the main constituents of *C. coggygria* leaves 60% methanol extract are quercetin rhamnoside (35.3 μg·mg^−1^), methyl gallate (31.2 μg·mg^−1^), methyl trigallate (29.7 μg·mg^−1^) and gallic acid (9.8 μg·mg^−1^). The quantification of identified compounds was performed using external standards. For the quantification of tannins and organic acids we used gallic acid and for the quantification of flavonols and their glycosides we used quercetin. Both standards showed good linearity. The following r^2^ values were obtained: gallic acid r^2^ = 0.9997, regression curve y = 374.62x − 47.676 and quercetin r^2^ = 0.9994, regression curve y = 181.53x + 96.522. LOD for gallic acid was 0.41 μg·mg^−1^ and LOQ 1.36 μg·mg^−1^. LOD for quercetin was 0.92 μg·mg^−1^ and LOQ 3.06 μg·mg^−1^.

## 3. Experimental Section

Powdered 60% methanol extract of *C. coggygria* Scop. leaves was supplied by Florex Ltd. (Pernik, Bulgaria), Batch No. 01, Date: 20 January 2014.

### 3.1. Bacterial Strains

Two collection *S. aureus* strains (CCM 4750/ATCC 4330 and CCM 4723/ATCC 29213) and 10 clinical *S. aureus* strains, isolated from atopic dermatitis and impetigo skin lesion swabs, were included into the study. The clinical strains were isolated at the Institute of Microbiology of the Medical Faculty Comenius University, and the University Hospital in Bratislava. For further characteristics of the strains see Table 1.

### 3.2. Antimicrobial Susceptibility Testing

Antimicrobial susceptibility to oxacillin, erythromycin, clindamycin, gentamicin, ciprofloxacin, cotrimoxazole, rifampicin, tetracyclin, chloramphenicol and mupirocin was detected by the disk diffusion method. The procedure and the interpretation of results were performed according to the EUCAST recommendations [26]. Commercial antibiotic disks were used (Oxoid, Basingstoke, Hampshire, UK).

### 3.3. Detection of Biofilm Formation

Biofilm production by the tested *S. aureus* strains was detected by microtiter plate technique according to Stepanović *et al.* [14]. Tryptose soy broth (Oxoid) supplemented by 1% glucose was used. The test wells of the sterile P-shaped microtiter plate contained 2 × 10^5^ bacteria in 200 μL of the broth. Biofilm was allowed to form for 24 h at 35 °C. Afterwards, the medium was removed from the wells and the biofilm was gently washed 3-times with PBS (pH 7.2), fixed with methanol, stained by crystal violet, the dye was dissolved in ethanol and the intensity of biofilm formation was measured as absorbance at 570 nm by an ELISA-reader (MRX Microplate Reader, Dynex Technologies, Chantilly, VA, USA).

### 3.4. Antimicrobial Activity Testing

Antimicrobial activity of *C. coggygria* leaves extract was tested by the broth microdilution method according to the EUCAST recommendations [26]. The dried powdered extract was dissolved in distilled water, filtration-sterilised and adjusted in Mueller-Hinton broth (Oxoid) to serial dilutions from 250 to 0.078 mg·mL^−1^. One hundred μL aliquots of the tested extract in particular concentrations were placed to the U-shaped sterile microtiter plate wells, and 10 μL aliquots of the tested bacterial strain suspension containing 5 × 10^6^ bacteria·mL^−1^ in Mueller-Hinton broth were inoculated into the wells with the tested extract. Mueller-Hinton broth without antimicrobial agents was used for bacterial growth control, and wells containing broth and serial dilutions of the extract were included as the sterility controls (negative controls). MIC was detected as the lowest concentration of the extract inhibiting the growth of bacteria. MBC was determined after inoculation of bacteria from wells with inhibited bacterial growth on antimicrobial-free agar medium plates. The bacterial growth was evaluated after overnight cultivation at 35 °C. The MBC was determined as the lowest concentration of the tested extract able to inactivate 99.9% of the tested bacterial inoculum. The tests were repeated in three independent runs.

### 3.5. Antibiofilm Activity Testing

Antibiofilm activity of extract was detected on 24 h biofilm prepared according to Stepanović *et* *al.* [14] on the surface of a sterile P-shaped polystyrene microtiter plate wells. After washing out the free bacterial cells, the extract was applied to biofilm and incubated for 24 h. The minimal biofilm inhibitory activity was detected visually as the lack of turbidity due to inhibition of bacterial growth. Afterwards, the medium with extract was gently removed, the wells were washed and extract-free medium was applied to the biofilms in wells. After 24 h cultivation, the vitality of bacteria was assessed by regrowth detection, *i.e.*, by observation of the presence or the lack of the medium turbidity [18]. The tests were performed in triplicates in three independent runs.

### 3.6. Phenolic Fingerprint. Identification of the Characteristic Constituents

The LC-MS analyses were performed on an Agilent 1260 Infinity LC System (Agilent Technologies, Santa Clara, CA, USA), equipped with a binary pump, an auto sampler, a column thermostat, and a diode array detector (DAD), coupled to a quadrupole-time of flight (6520 Accurate-Mass QTOF) instrument equipped with an orthogonal ESI source (Agilent Technologies). HPLC separation of *C. coggygria* extract was carried out on a Kromasil C18 column (150 mm × 4.6 mm, 5 μm, Sigma-Aldrich, Munich, Germany) at 35 °C and a flow rate of 0.4 mL/min. Water (adjusted to pH 3.1 with HCOOH/NH_4_HCO_2_) and MeCN were used as mobile phase A and B respectively. The following gradient program was used: 10% B (20 min), 20% B (25 min), 60% (50 min), 95% (62 min) and 10% (70 min). The ESI ion source parameters were as follows: capillary voltage: 3.5 kV, nebulizer: 40 psi (N2), dry gas flow: 10 L/min (N_2_), and dry temperature: 300 °C. The mass spectrometer was operated in an autoMS2 mode where each negative ion MS scan (*m*/*z* 100–3000, average of four spectra) was followed by MS^2^ scans (*m*/*z* 100–3000, average of four spectra, isolation window of 4 amu, collision energy 20 eV) of the two most intense precursor ions. Ions were excluded from analyses for 0.5 min after two MS2 spectra had been acquired. Nitrogen was used as collision gas. Phenolic compounds were identified by comparing their UV and mass spectra with literature and authentic standards when available and by measuring accurate *m*/*z* values [27].

### 3.7. Qualitative Determination of Constituents

The quantitative determination of phenolic compounds in *C. coggygria* methanol extract was provided by the method of external standards. We used quercetin for determination of quercetin and myricetin derivatives, gallic acid for the quantification of gallic acid and its derivatives, quinic acid and protocatechuic derivative (see Table 1). Chromatographic standards were purchased from Sigma-Aldrich. The examinations of secondary metabolites in *C. coggygria* methanol extract were performed in triplicate. The quantitative results were calculated from calibration curves, expressed as mean values and standard deviation (SD).

## 4. Conclusions

The results obtained in the present work reveal a high antibacterial and anti-biofilm efficacy of *C. coggygria* leaves methanol extract against collection and clinical *S. aureus* strains. The extract showed bactericidal activity against all tested *S. aureus* strains, including polyresistant strains, and eradicated bacteria in already established 24 h biofilm. The polyphenolic fingerprint performed by LC-MS/MS revealed the presence of tannins and flavonoids as the main secondary metabolites in leaves of which quercetin rhamnoside, methyl gallate, methyl trigallate and gallic acid were the most abundant in the tested methanol extract. Based on the obtained results, *C. coggygria* leaves 60% methanol extract may be ranked among natural agents with promising therapeutic potential, especially for supportive local treatment of staphylococcal infections. Our results can be considered as a unique contribution to the research of the natural antimicrobial agents.
